# Calculating air volume fractions from computed tomography images for chronic obstructive pulmonary disease diagnosis

**DOI:** 10.1371/journal.pone.0231730

**Published:** 2020-04-16

**Authors:** Chun-Chao Chuang, Ying-Hsiang Chou, Shin-Lei Peng, Jou-Erh Tai, Shan-Chih Lee, Yeu-Sheng Tyan, Cheng-Ting Shih

**Affiliations:** 1 Department of Medical Imaging and Radiological Sciences, Chung Shan Medical University, Taichung, Taiwan; 2 Department of Medical Imaging, Chung Shan Medical University Hospital, Taichung, Taiwan; 3 Department of Radiation Oncology, Chung Shan Medical University Hospital, Taichung, Taiwan; 4 Department of Biomedical Imaging and Radiological Science, China Medical University, Taichung, Taiwan; Clinic for Infectious and tropical diseases, Clinical centre of Serbia, SERBIA

## Abstract

Quantitative evaluation using image biomarkers calculated from threshold-segmented low-attenuation areas on chest computed tomography (CT) images for diagnosing chronic obstructive pulmonary diseases (COPD) has been widely investigated. However, the segmentation results depend on the applied threshold and slice thickness of the CT images because of the partial volume effect (PVE). In this study, the air volume fraction (AV/TV) of lungs was calculated from CT images using a two-compartment model (TCM) for COPD diagnosis. A relative air volume histogram (RAVH) was constructed using the AV/TV values to describe the air content characteristics of lungs. In phantom studies, the TCM accurately calculated total cavity volumes and foam masses with percent errors of less than 8% and ±4%, respectively. In patient studies, the relative volumes of normal and damaged lung tissues and the damaged-to-normal RV ratio were defined and calculated from the RAVHs as image biomarkers, which correctly differentiated COPD patients from controls in 2.5- and 5-mm-thick images with areas under receiver operating characteristic curves of >0.94. The AV/TV calculated using the TCM can prevent the effect of slice thickness, and the image biomarkers calculated from the RAVH are reliable for diagnosing COPD

## Introduction

Chronic obstructive pulmonary disease (COPD) is a type of progressive and obstructive lung disease involving emphysema and chronic bronchitis. In patients with COPD, airway structures are remodeled by severe destruction or inflammation of alveoli and small airways, resulting in the decrease of lung elasticity, hyperinflation, and air trapping [1–3]. Consequently, lung capacity is drastically reduced and patients experience chronic cough, dyspnea, and poor airflow. COPD has high mortality: it was the fourth leading cause of death in 2015, and its mortality rate is increasing [4]. Therefore, accurate and rapid COPD diagnosis is crucial in clinical medicine.

The pulmonary function test (PFT), a standard procedure used to diagnose and evaluate COPD, includes measuring the forced expiratory volume in 1 s (FEV1) and the ratio of FEV1 to the forced vital capacity (FVC; FEV1/FVC ratio) [5–7]. The current definition of COPD per Global Initiative for Chronic Obstructive Lung Disease (GOLD) is an FEV1/FVC ratio of <0.7; moreover, COPD severity is graded using the FEV1 values [7]. However, the measured results are patient dependent and typically biased, particularly in older patients [8]. To improve the quality of evaluation of COPD, quantification methods using computed tomography (CT) images have been widely investigated in the last three decades [9–21]. These methods are mostly based on detecting abnormal air content in lungs as low-attenuation areas on binary segmentation as image biomarkers for COPD diagnosis and evaluation [14,16,19,20]. Percent emphysema (PE), defined as a percentage of the segmented volume to the total lung volume, is calculated using a threshold between −910 and −950 HU [10,11,13,15,17]. Parametric response mapping applies two thresholds of −950 and −856 on the CT images from full inspiration and normal expiration scans, respectively, to calculate relative volumes (RVs) of emphysema and functional small airways disease [18,21]. However, CT images have a limited spatial resolution, with a voxel size (0.125–1.25 mm^3^) approximately 30–300 times larger than the average size of an alveolus (0.0042 mm^3^) [12]. The CT number of an air space formed by several damaged alveoli could vary with the voxel size because of the partial volume effect (PVE) [22]. Therefore, the threshold applied for segmentation affects the calculated RV and should be adjusted according to the imaging parameters, particularly slice thickness, to ensure comparable results. The threshold values applied in recent studies were determined based on macroscopic pathological features of emphysema [9,10,23], quantitative histological comparisons [24], or correlations between the segmented RVs and PFT results [13,17,19]. Nevertheless, the relationship between the threshold and slice thickness remains unclear; the relationship between the segmented RV and PFT results has been proven to be nonlinear [20]. Moreover, binary segmentation using a threshold to separate air or tissues in CT images could be problematic. Image voxels in the lungs typically cover mixtures of air and tissues with different proportions.

It has been known that the COPD affects the air contain in lungs. In this study, the air volume fraction (AV/TV) in lungs was calculated from CT images using a two-compartment model (TCM), and image biomarkers derived from the AV/TV were used as novel image biomarkers for COPD diagnosis. Comparing with volumetric threshold segmentation, the TCM considers lung tissues are comprising air and lung parenchyma; CT images can be converted to AV/TV maps on the basis of the partial linear attenuation coefficients of air in each image voxel, that prevent the influence from the PVE. Relative air volume histograms (RAVHs) and image biomarkers were further calculated from the AV/TV maps to characterize the air content in the patient’s lungs. The accuracy of binary segmentation and the TCM in calculating AV/TV from CT images was evaluated and compared using phantom studies. The performance of the PE and image biomarkers from the RAVH for COPD diagnosis was assessed through patient study. Moreover, the effect of slice thickness in calculating AV/TV and COPD diagnosis was evaluated.

## Materials and methods

### Calculating AV/TV from CT images by using TCM

In this study, the TCM was applied to calculate AV/TV of lungs from CT images. The TCM can nullify the effect of the PVE and energy-spectrum dependence between scans, enabling accurate calculation of the volumes of subcomponent materials from CT images if a tissue can be simplified to comprise two subcomponent materials. The concept of the TCM is described as follows [22]: The TCM considers an image voxel to be filled with a mixture *M*, which consists of two submaterials, *a* and *b*, with specific volume fractions. If the CT numbers of the submaterials are known, the volume fraction of the submaterial *a* in the voxel can be calculated using the TCM as follows:

$$v_{a}=\frac{HU_{M}-HU_{b}}{HU_{a}-HU_{b}}$$ (1)

where *HU*_*a*_, *HU*_*b*_, and *HU*_*M*_ are the CT numbers of *a*, *b*, and *M*, respectively. According to elemental composition reported previously [25,26], tissues included in common chest CT images, such as lung parenchyma, muscle, heart, blood, and aorta, have similar physical density, electron density, and effective atomic numbers, with the maximum difference of <2.5% than that of the lung parenchyma (see S1 Table). Therefore, these tissues have a similar CT number and can be represented by one of them, and image voxels in lungs can be considered to include a mixture of air and a representative tissue. Therefore, AV/TV can be calculated from the CT numbers of lungs using the TCM as follows:

$$AV/TV=\frac{HU_{L}-HU_{T}}{HU_{A}-HU_{T}}$$ (2)

where *HU*_*T*_, *HU*_*A*_, and *HU*_*L*_ were the CT numbers of the representative tissue, air, and the lung to be evaluated, respectively. The CT number of air can be measured from the CT images by applying the region of interest (ROI) outside the patient’s body. Because directly calculating the CT number of the representative tissue from the CT images is difficult, muscle and blood were used as the representative tissue and submaterial pairs of air–muscle and air–blood were used to convert chest CT images to AV/TV maps.

### Lung equivalent phantoms

Lung equivalent phantoms, including cavity and foam phantoms, were used to simulate the anatomical and pathological structures appearing in the chest CT images of COPD patients. Five cavity phantoms were used to mimic air cavities in patients with emphysema with their sizes larger than that of the voxel. The cavity phantoms were printed using a 3D printer (Object 500 connex 3, Stratasys, Eden Prairie, Minnesota) and an ultraviolet-curable polyurethane (PU) with a density of 1.169-g/cm^3^. Each cavity phantom comprised a cavity cube and pair of caps. The cavity cube dimensions were 30 × 30 × 20 mm^3^, with a 5-mm-thick wall. The caps closely covered the top and bottom of the cube to form a closed space. In the five cavity cubes, septa with widths of 3, 2, 0.7, 0.7, and 1 mm, with spacing (cavity widths) of 1.9, 2, 2.8, 3, and 4 mm dividing the closed spaces into small cavities. The total cavity volumes, equal to the total air volumes in the phantoms, were 4.4, 5.6, 6.04, 6.6, and 6.8 cm^3^, with the numbers of the small cavities being 4, 4, 8, 6, and 4, respectively (see S1 Fig).

Three 5 × 5 × 5-cm3 cubic foam phantoms with 10, 20, and 60 pores per inch (PPI) were used to simulate alveoli and small air spaces formed by damaged alveoli, with their size similar to or smaller than that of the voxel size. The foams were composed of polyurethane (PU) with a raw material density of 1.12 g/cm3. The masses of the foams with 10, 20, and 60 PPIs were 2.82, 3.02, and 2.67 g, respectively. The pores in the foams were distributed uniformly.

### Data analysis and evaluation of phantom and patient studies

**Fig 1:**
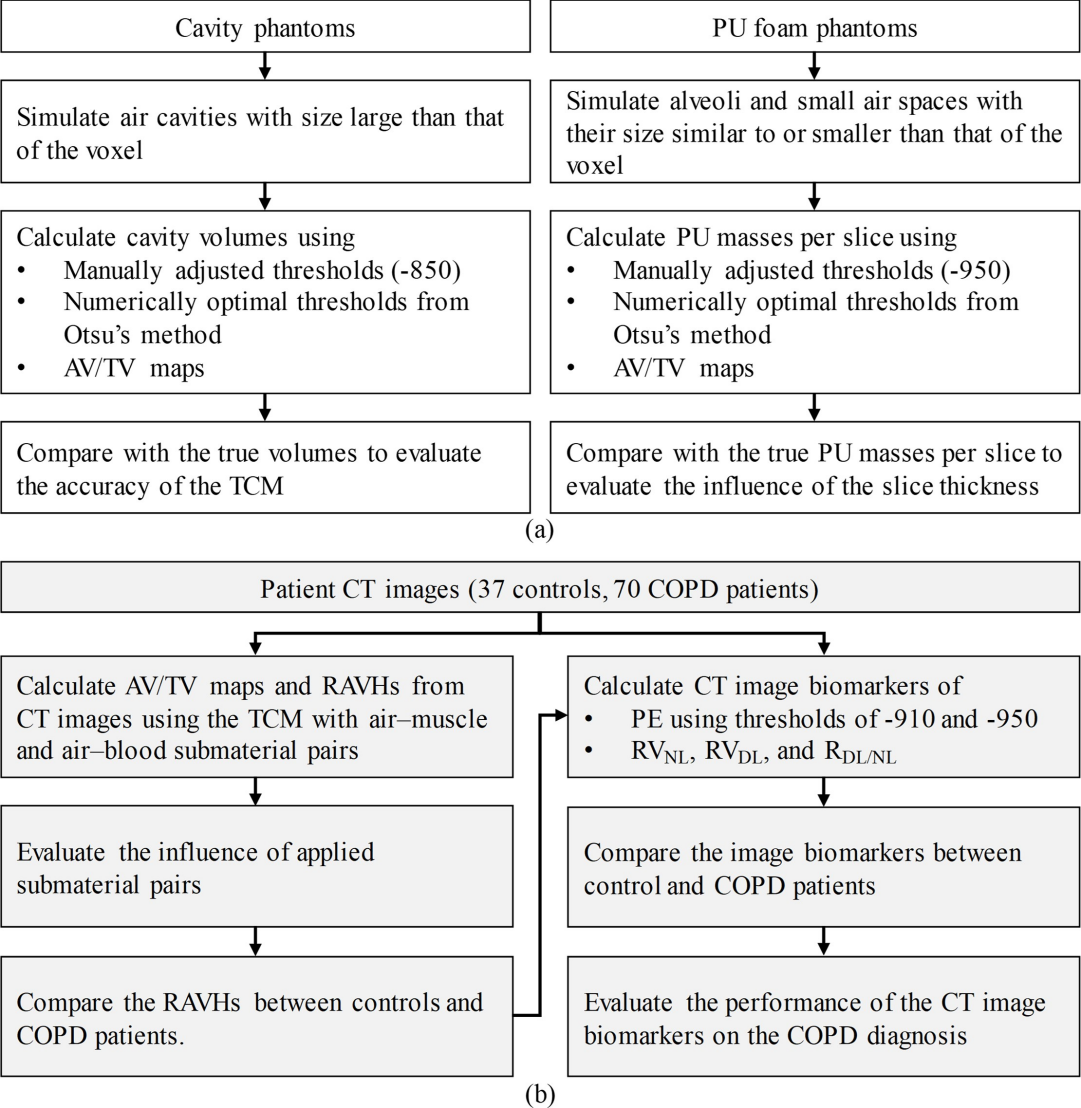
Block diagrams that represents the evaluation procedure of the (a) phantom and (b) patient studies performed in this study.

The cavity and foam phantoms were used to validate the accuracy of the TCM in calculating AV/TV from CT images. The CT images of the phantoms were acquired using a multidetector row CT scanner (Optima CT660, GE Healthcare, Milwaukee, WI) with a routine protocol of a tube voltage of 120 kVp in the helical mode. The tube current was adjusted using the automatic exposure control technique. During the multidetector CT scan, the phantoms were fixed on a solid-water slab. The voxel size of the reconstructed image was 0.47 × 0.47 × 0.05 mm^3^. The effect of the submaterial pair and slice thickness on the calculation of AV/TV using the TCM and the performance of AV/TV in the diagnosis of COPD were assessed using patients’ CT images. The CT and PFT results of both the controls and patients with COPD were retrospectively retrieved and assessed. This retrospective study was conducted at the Chung Shan Medical University Hospital under permission of the Institutional Review Board of the Chung Shan Medical University Hospital (IRB No. CS2-19091), which approved this study waived the need to obtain informed consent. The span between the CT scan and PFT was maintained at <1 month to ensure the lung capacity of the patients did not significantly change. The chest CT images of all participants were retrieved through a picture archiving and communication system; these images were acquired during full inspiration by using the same scanner used in the phantom studies with a routine protocol of a tube voltage of 120 kVp in helical mode. The images with slice thicknesses of 2.5 and 5 mm were separately reconstructed using a default body filter. Fig 1 shows block diagrams that summarize the evaluation procedures of phantom and patient study used in the study. The detailed procedures were described in the following sections.

### Evaluation of the accuracy of the TCM in calculating AV/TV from CT images

In the cavity phantom study, the total cavity volumes were calculated using binary segmentation and the TCM with the air–PU submaterial pair. Binary segmentation was performed using a manually adjusted threshold value of −850 and a numerically optimal value derived using Otsu’s method [22,27]. The total cavity volumes were calculated by multiplying the number of the segmented voxels with the voxel size. The optimal threshold values for each cavity phantom were separately determined. For the TCM, the CT numbers of the air–PU submaterial pair were measured from the phantom CT images using ROIs. The phantom CT image volumes were converted to AV/TV by using Eq (2) with the measured CT numbers of the submaterials, and the total cavity volumes were calculated by multiplying the sum of the AV/TV values with the voxel size. The calculated total cavity volumes were compared with the true volumes.

### Evaluation of the influence of the slice thickness of CT images on calculating AV/TV and RAVH

The effect of slice thickness on AV/TV calculations using the TCM was also evaluated in the foam phantom study. However, the true air volume in the foams could not be directly obtained as a reference standard. Furthermore, the CT number of the bottom of the foams can be affected by the supporting solid-water slab because of the PVE. Nevertheless, these foams comprised only PU and air, consistent with the TCM. After the total PU volume was obtained, total air volume of the foam could be derived. In addition, the masses of the foams and physical density of the PU were known. Therefore, the foam phantom study was performed by evaluating the PU masses in a single coronal slice of the foam. The coronal CT images of the foams were reconstructed using the multiplanar reconstruction technique with slice thicknesses of 0.47, 0.94, 1.88, 2.82, and 4.7 mm. The true mass of the PU per slice of the foam was calculated as a fractional mass by multiplying the weighted total mass of the foam with the ratio of the slice thickness to the width of the foam (5 cm). The slices reconstructed from the middle region of the foams were used to calculate the PU mass per slice. For binary segmentation, a fixed threshold of −950 and an optimal threshold from Otsu’s method were applied to the CT images, and the PU mass per slice was calculated by multiplying the number of the segmented voxels with the known voxel size and the physical density of the PU. The optimal thresholds from Otsu’s method for each foam and slice thickness were separately determined. For the TCM, the CT number of the submaterials of air and the PU were measured from the CT images of the raw PU material by using the ROIs. The CT images of the foams were converted to PU volume fraction maps using Eq (2) with the measured CT numbers of the submaterials. The PU mass per slice was then calculated by multiplying the sum of the PU volume fraction map with the known voxel size and the physical density of the PU. The PU mass per slice calculated using binary segmentation and the TCM was compared with the calculated fractional mass.

In addition, the effect of the image voxel and anatomical structure sizes on calculation of RAVH was also evaluated using the foam phantoms. The RAVHs with bin centroids from 1.25% to 98.75% AV/TV and a step size of 2.5% AV/TV were calculated from the 0.47-, 0.94-, 1.88-, 2.82-, and 4.7-mm-thick coronal images of the foams with PPIs of 10, 20, and 60. The RVs of the 98.75% AV/TV bin (RV_98.75%_) were acquired from the RAVHs and were compared.

### Conversion of CT images to AV/TV maps and calculation of RAVHs

**Fig 2:**
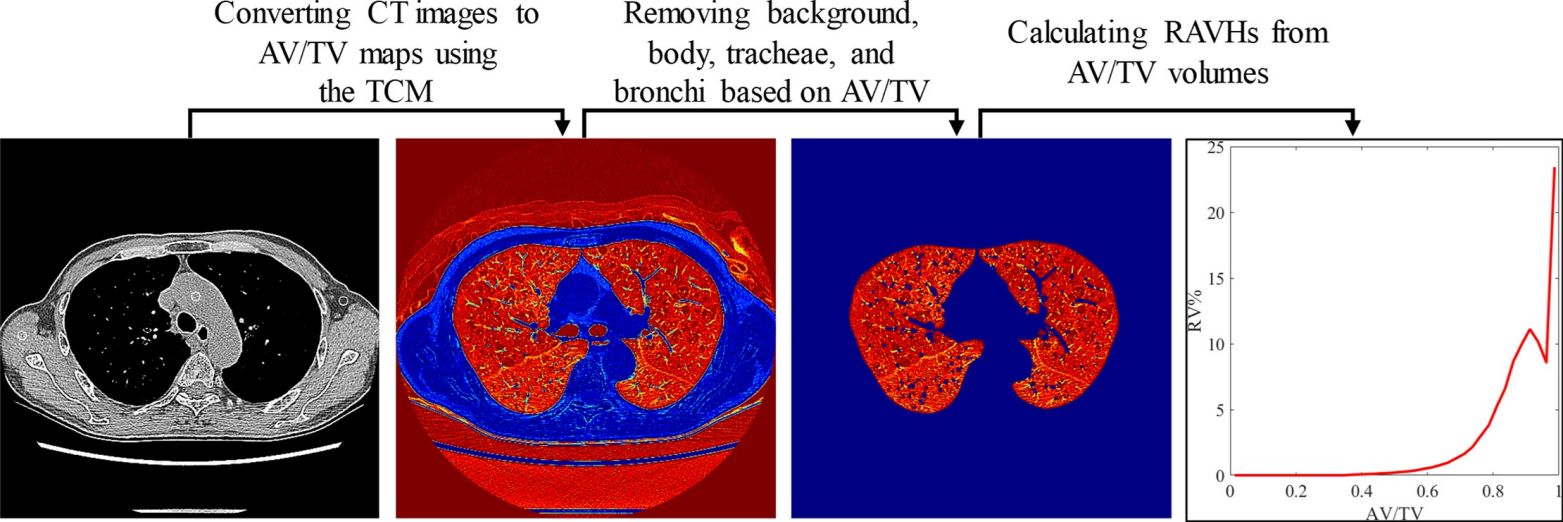
Processing procedures of patient CT images. The CT images were converted to AV/TV maps using the TCM with the measured CT numbers of muscle and air. The background, body, tracheae, and bronchi were segmented and removed from the map based on the AV/TV value. Relative air volume histograms were calculated from the AV/TV volumes.

The chest CT image volumes of the participants were converted to AV/TV by using the TCM with air–muscle and air–blood submaterial pairs. The processing procedures of patient CT images are shown in Fig 2 and as follows. For each patient, the CT numbers of muscle, blood, and air were manually measured from their CT images using circular ROIs defined in uniform regions by a radiologist with 10 years of clinical experience. The ROIs for muscle and blood were specifically defined around or between ribs and in the aorta or ventricle, respectively [25]. The measured CT numbers of muscle and blood were compared using paired-sample *t* tests. After the conversion, lung masks were generated from the AV/TV using a threshold of 1% AV/TV to segment the lung regions. Furthermore, a region growing process with a seed of 100% AV/TV defined in the trachea and a growth stopping criterion of a 95% AV/TV was applied to remove the trachea and connected bronchi. To parameterize the air content characteristics of the lungs, the mean AV/TV values and RAVHs with bin centroids from 1.25% to 98.75% AV/TV and a step size of 2.5% AV/TV were calculated from AV/TV.

### Evaluation of the performance of the AV/TV maps and RAVHs on the COPD diagnosis

The mean AV/TV was calculated using the TCM with different submaterial pairs were compared using a Bland–Altman plot with 95% confidence intervals. The mean AV/TV value, as well as the RVs of specific AV/TV bins acquired from the RAVH, were used as image biomarkers to distinguish patients with COPD from control participants. For comparison, the image biomarkers of the PE were calculated by applying thresholds of −910 (PE_−910_) and −950 (PE_−950_) on the CT images after removing the trachea and connected bronchi using the lung masks. Comparisons between the image biomarkers of the control and COPD groups were conducted using the independent-sample *t* test. To assess the diagnostic performance of the image biomarkers, receiver operating characteristic (ROC) curves and area under the ROC curves (AUCs) were determined [28], and sensitivity and specificity were calculated. As the area under the RUC curve is closer to 1, the better the diagnostic performance of the applied image biomarkers. All statistical analyses were performed on MedCalc (version 15.8; MedCalc Software, Ostend, Belgium) with statistical significance defined at a *P* value of <0.05.

## Results

**Fig 3:**
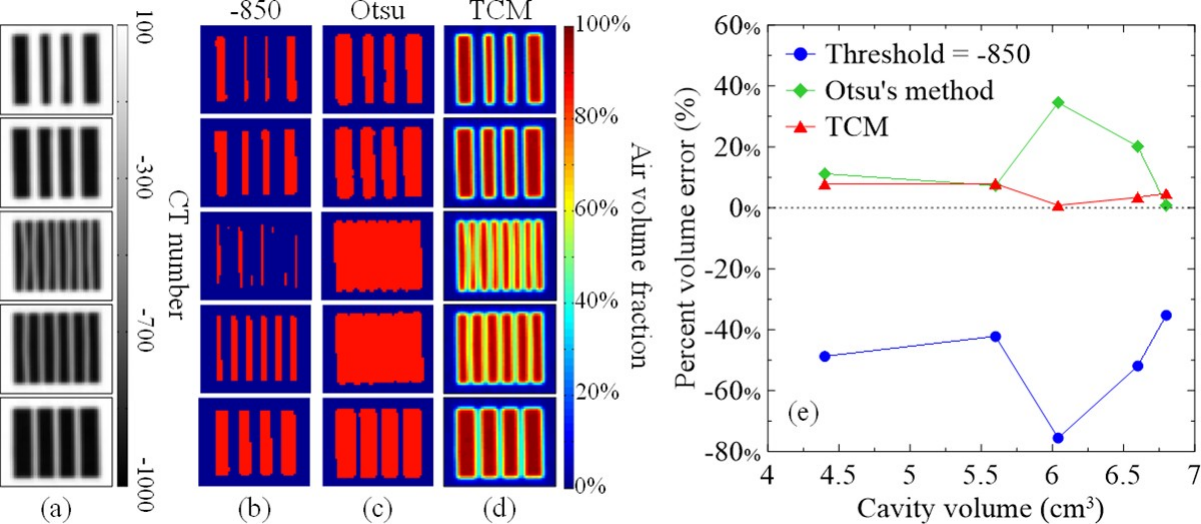
(a) CT images of cavity phantoms. (b) and (c) present binary segmentation results from (a) using the threshold of −850 and the thresholds from Otsu’s method. (d) AV/TV maps calculated from (a) using the TCM with an air–PU submaterial pair. (e) Percent errors between the segmented and TCM-calculated total cavity volumes and the true volumes as functions of the true volumes.

Fig 3(A) illustrates the CT images of the cavity phantoms. The septa and cavity walls were printed using the same materials to ensure a similar CT number. Septa thicker than 2 mm (first and second rows) presented CT numbers similar to those of the wall and could be clearly distinguished from the air background. However, the septa thinner than 2 mm (third to fifth rows) presented CT numbers lower than those of the wall and were blurred because of the PVE. Fig 3(B) and 3(C) illustrate the binary segmentation results from Fig 3(A). Binary segmentation using the thresholds of −850 can separate most of the cavities from the CT images; however, parts of the cavities narrower than 2 mm disappeared (Fig 3(B), third row). By contrast, cavity volumes segmented using the thresholds derived from Otsu’s method were relatively larger than those segmented using the threshold of −850. Nevertheless, cavities and septa narrower than 1 mm could not be separated and segmented. The optimal thresholds derived from Otsu’s method for the five cavity phantoms (Fig 3(C)) from top to bottom were −386.5, −412.5, −310, −370.6, and −447.6. For the TCM, the CT numbers of the PU and air were 113.4 and −999.5, respectively. The AV/TV maps calculated using the TCM from Fig 3(A) preserved all septa and cavities, as shown in Fig 3(D). Fig 3(E) shows the percent errors between the calculated total cavity volumes and the true volumes as functions of the true volumes. Binary segmentation using the thresholds of −850 underestimated the total cavity volumes by >35% in all cavity phantoms. Otsu’s method accurately calculated the total cavity volumes of 4.4, 5.6, and 6.8 cm^3^ with errors of <12% but overestimated the volumes of 6.04 and 6.6 cm^3^ by >20% because of the inseparable septa and cavities narrower than 1 mm. In contrast to binary segmentation, the TCM accurately calculated total cavity volumes of all cavity phantoms with errors of <8%, indicating that the TCM did not depend on the size of the structure.

**Fig 4:**
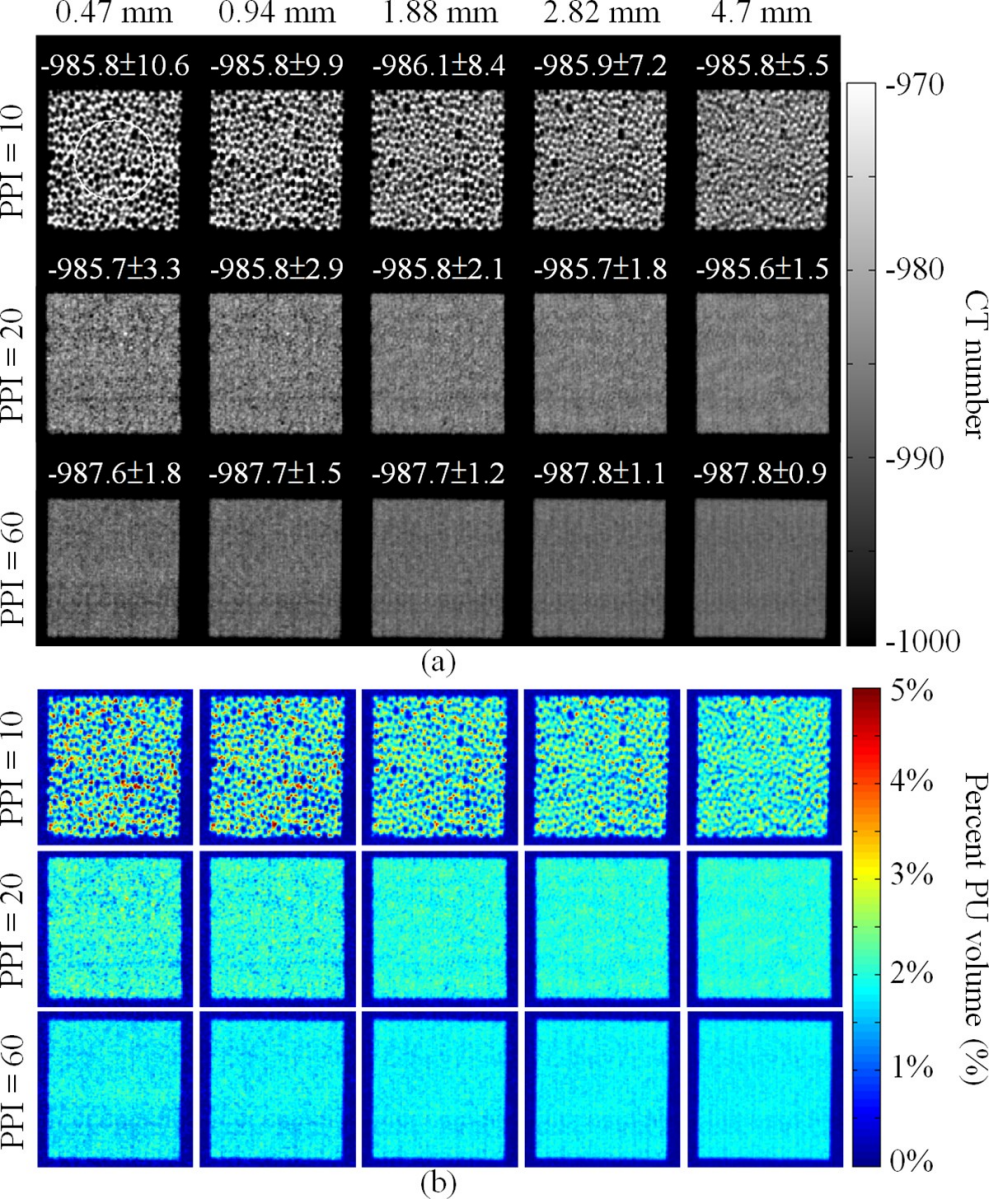
(a) CT images of the foams with slice thicknesses of 0.47, 0.94, 1.88, 2.82, and 4.7 mm with addressed mean CT numbers of the foams. (b) PU volume fraction maps calculated from (a) using the TCM with an air–PU submaterial pair. The white circle in (a) indicates the location of the ROI for the calculation of the mean CT numbers.

**Fig 5:**
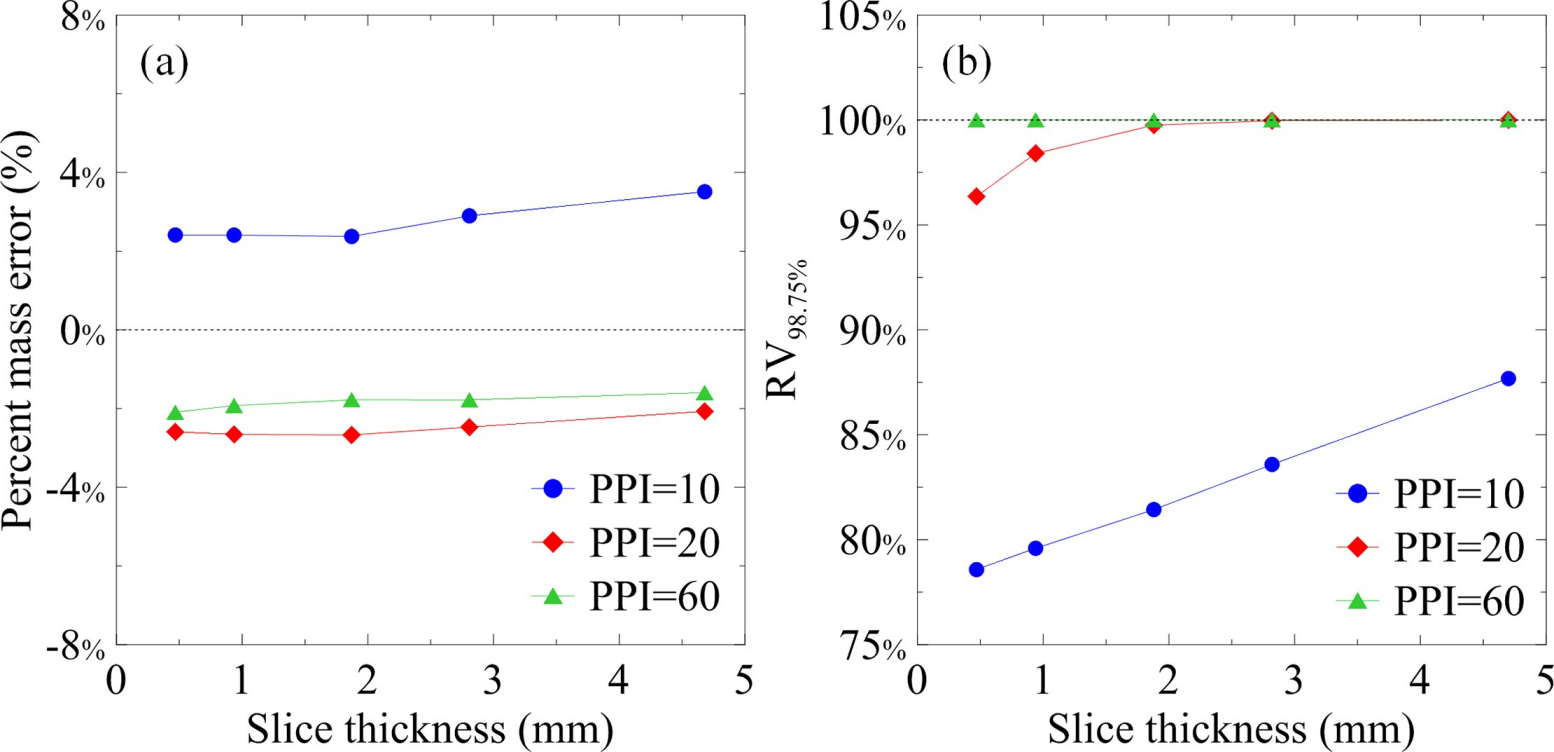
(a) Percent errors between the weighted and TCM-calculated PU masses per slice of the foam phantoms as a function of the slice thickness. (b) RV_98.75%_ values acquired from the RAVHs of the foams with the PPIs of 10, 20, and 60 as a function of the slice thickness.

Fig 4(A) shows the CT images of the foams with slice thicknesses of 0.47, 0.94, 1.88, 2.82, and 4.7 mm. The means and standard deviations of the CT numbers of the foams were measured using circular ROIs and were labeled in the images. The mean CT numbers of the foams of different PPIs and slice thicknesses were similar. However, the visibility of the pore structures was reduced and the standard deviations of the CT numbers of the foams decreased as the PPIs or slice thicknesses increased, reflecting the blurring of the foams as uniform objects because of PVE. In binary segmentation, the volumes of the segmented PU structures increased as the PPIs or slice thicknesses increased. In foams with PPIs of 20 and 60, few or no pores were separated by binary segmentation (see S2 Fig). Fig 4(B) shows the PU volume fraction maps calculated from Fig 4(A) using the TCM. The PU volume fractions varied with the PPIs and were <5% for all foams. The segmented PU structure maps and PU volume fraction maps were used to calculate the PU mass per slice. Compared with the weighted results, the PU masses per slice calculated using binary segmentation were overestimated by >1000% for all foams and all slice thicknesses. Fig 5(A) shows the percent errors between the weighted and TCM-calculated PU masses per slice of the foam phantoms as functions of the slice thickness. In contrast to binary segmentation, the TCM was insensitive to slice thickness and pore size. The percent errors of the TCM-calculated PU masses per slice were less than ±4% for all foams and all slice thicknesses. Fig 5(B) shows the RV_98.75%_ values acquired from the RAVHs of the foams with PPIs of 10, 20, and 60 as functions of the slice thickness. The change in RV_98.75%_ with change in slice thickness was dependent on the PPI of the foam. The RV_98.75%_ of the foams with 10 and 20 PPIs were increased with the slice thickness. However, the RV_98.75%_ values of the foam with the PPI of 60 saturated at 100% across all slice thicknesses. The RAVH calculated from CT images was dependent on the imaging structure size relative to the voxel size.

Table 1 shows the demographic data and pulmonary function of participants. The control group included 37 participants confirmed to be COPD-free based on medical history, medical images, and PFT. The CT scans and PFTs of the control participants were performed for regular health examination. Details of 75 participants who were confirmed to have COPD based on PFT with the GOLD criteria and medical imaging were included initially. Five patients were excluded because of the use of contrast medium and presence of intense image artifacts. Finally, the COPD group included 70 participants.

**Table 1:** Demographic data and pulmonary function of participants.

Parameter	Control (*n* = 37)	COPD (*n* = 70)	*P* value
Age (y)	51.6 ± 11.9	57.5 ± 12.3	0.018
Percentage of women	56.8%	32.9%	0.017
BMI	26.6 ± 5.5	25.5 ± 3.8	0.434
FEV1/FVC (%)	85.3 ± 7.46	44.0 ± 15.3	<0.0001

The mean CT numbers of muscle, blood, and air measured for the 107 patients were respectively 50.7 ± 10.0, 46.2 ± 17.0, and −999.3 ± 6.3 in the 2.5-mm-thick images and were 52.0 ± 9.7, 50.3 ± 11.9 and -996.8 ± 3.1 in the 5-mm-thick images. Significant differences were found between the mean CT numbers of muscle and blood in both 2.5- and 5-mm-thick images (*P* < 0.05). By contrast, the mean CT numbers of air from 2.5- and 5-mm-thick images were not significantly different (*P* = 0.43). The mean CT numbers of the submaterials were applied to calculate AV/TV using the TCM with air–muscle and air–blood submaterial pairs. Although the mean CT numbers of the submaterials were significantly different, the Bland–Altman plots show the mean AV/TV values of the lungs calculated for the 71 patients using the air–blood pair were comparable to those calculated using the air–muscle pair with a mean AV/TV difference of less than ±1% in the 2.5- and 5-mm-thick images (see S3 Fig). Based on the results, AV/TV calculated using the air–muscle pair were investigated in the subsequent studies.

**Fig 6:**
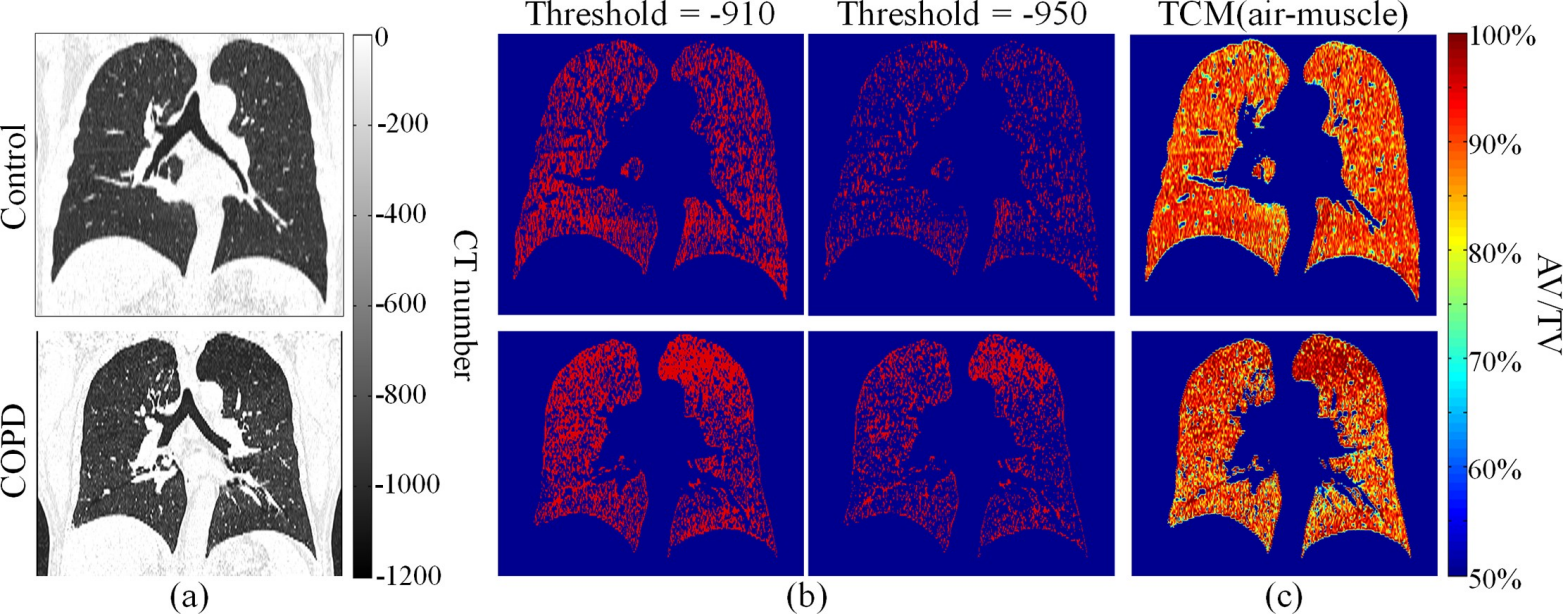
(a) Chest CT images of a control participant (top) and a patient with (bottom). (b) Binary segmentation results (a) using thresholds of 910 and 950. (c) AV/TV maps calculated (a) using the TCM with an air–muscle submaterial pair.

Fig 6(B) and 6(C) show the binary segmentation results and TCM-calculated AV/TV maps from the CT images of a control participant and a patient with COPD (Fig 6(A)), respectively. In binary segmentation, low-attenuation areas segmented from the images of the patient with COPD were larger than those segmented from the images of control participant across both thresholds. The segmented low-attenuation areas increased as the applied threshold increased. On the AV/TV maps, the mean AV/TV of the control participant and patient with COPD was 84.7% and 84.5%, respectively. It can be observed that the upper lobe of the left lung of a COPD patient presented as a low-attenuation area on binary segmentation and a relatively higher air content region on the AV/TV map.

**Fig 7:**
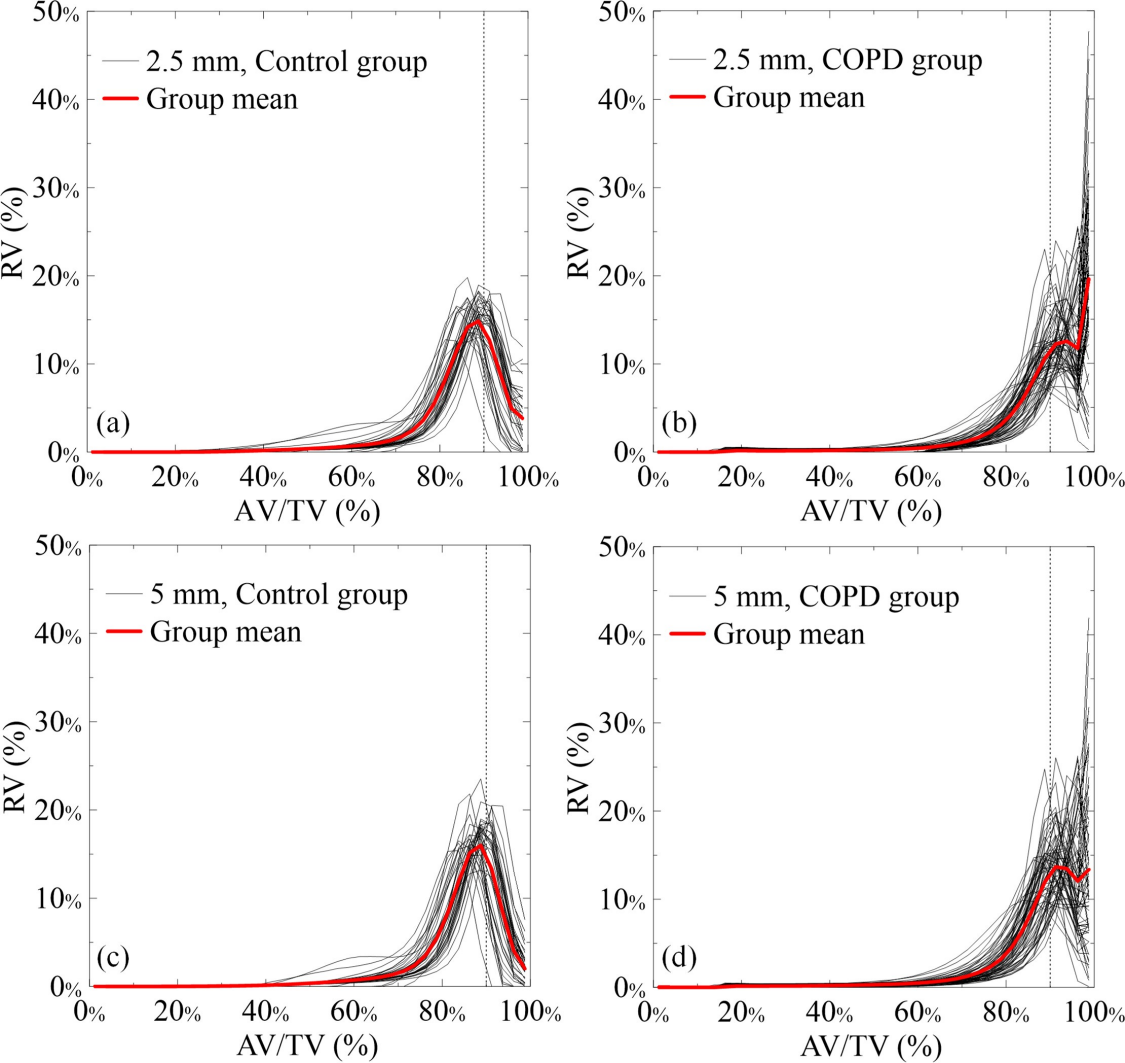
(a) and (b) RAVHs of the control and COPD groups calculated from the 2.5-mm-thick CT images, respectively. (c) and (d) RAVHs of the control and COPD groups calculated from the 5-mm-thick CT images, respectively. The group-mean RAVHs (red bold lines) are shown.

Fig 7 shows the RAVHs of the control participants and patients with COPD calculated from the 2.5- and 5-mm-thick CT images with addressed group-mean RAVHs. In the control group, the RAVHs presented as a normal distribution with the peak located around the 88.75% AV/TV bin in both slice thicknesses. In comparison with the 2.5-mm-thick images, the mean RAVH from the 5-mm-thick images presented a lower RV_98.75%_, revealing monotonous CT numbers by the PVE. The RVs of the 88.75% AV/TV bin (RV_88.75%_) of the mean RAVH from the 2.5- and 5-mm-thick images were 14.85% and 15.98%, respectively. In contrast, the RAVH of the COPD group presented two peaks: one around the 88.75% AV/TV bin and the other at the 98.75% AV/TV bin. As compared with the control group, the RV_88.75%_ was 4.3% lesser and the RV_98.75%_ was 15.8% higher in the mean RAVH of the COPD group. When the slice thickness increased to 5 mm, the RV_98.75%_ peak of the COPD group lowered. Compared to that with the control group, the RV_88.75%_ decreased by 4.1% and the RV_98.75%_ increased by 11.3% in the mean RAVH of the COPD group. According to the aforementioned results, indices were computed from the RAVHs as image biomarkers for differentiating COPD from the control group, including a total RV of the 81.25% to 91.25% AV/TV bins representing the RV of the normal lung (RV_NL_), the RV_98.75%_ representing the RV of the damaged lung (RV_DL_), and the RV_DL_/RV_NL_ ratio (R_DL/NL_) representing the ratio of the damaged-to-normal lung volume.

In comparison of the image biomarkers between groups, significant differences were found between all image biomarkers of the control and COPD groups in both slice thicknesses (*P* < 0.001) (see S4 Fig). However, the differences between the mean AV/TV values of the control and COPD groups were <5% across both slice thicknesses. When the slice thickness increased from 2.5 mm to 5 mm, the PE_-910_, PE_-950_, RV_DL_, and R_DL/NL_ decreased, the RV_NL_ increased for both groups, and the differences between the groups decreased. These changes were a result of the decreased number of voxels, with CT numbers similar to the CT number of air.

**Fig 8:**
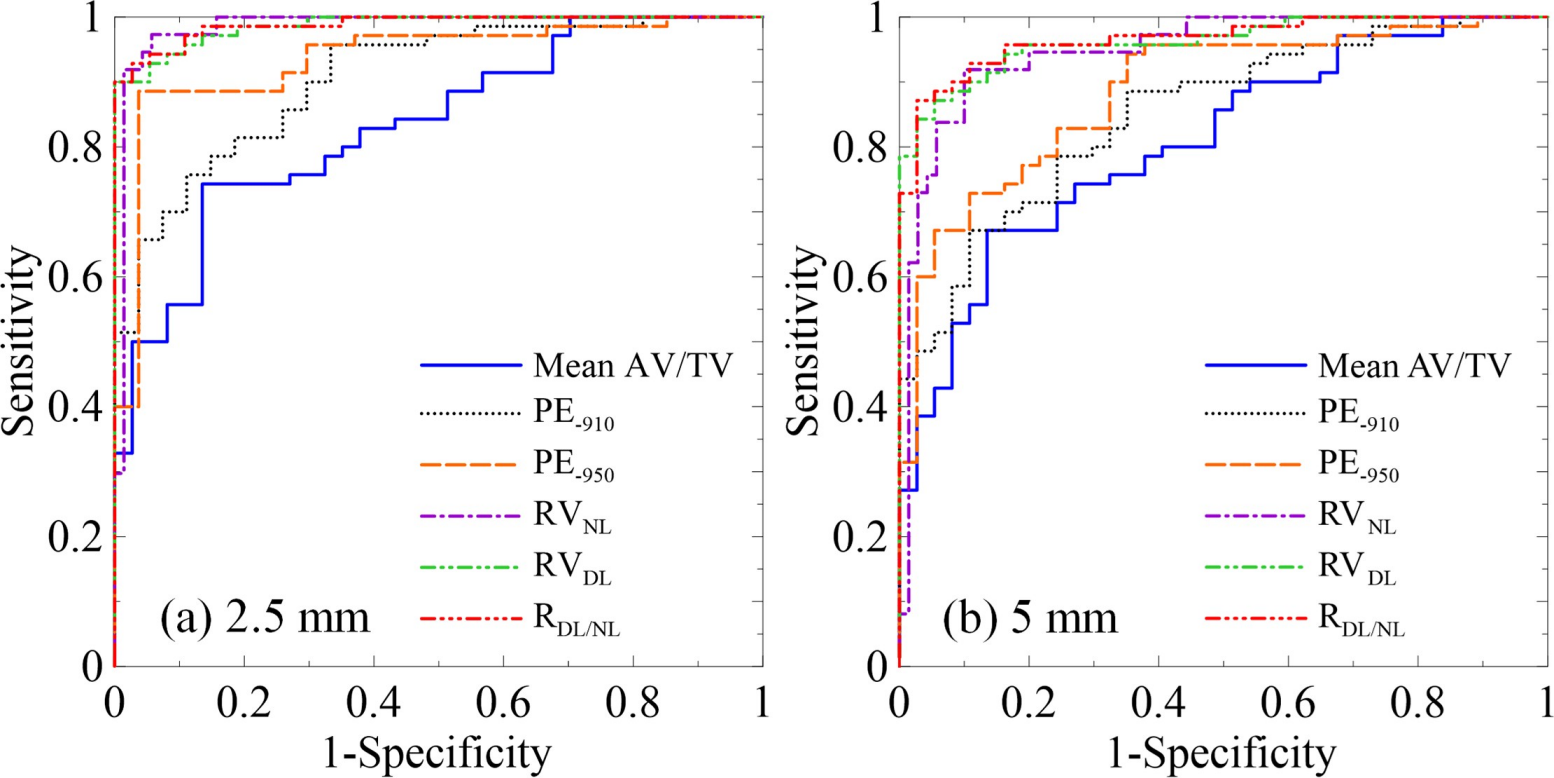
ROC curves show differentiation between control and COPD groups using the image biomarkers of mean AV/TV, PE_-910_, PE_-950_, RV_NL_, RV_DL_, and R_DL/NL_ from (a) 2.5- and (b) 5-mm-thick CT images. Areas under the curves, sensitivity, and specificity of the image biomarkers are summarized in Table 2.

Fig 8 shows the ROCs of the studied image biomarkers from the 2.5- and 5-mm-thick images. The AUC, sensitivity, and specificity are summarized in Table 2. In the 2.5-mm-thick images, the PE_-950_, RV_NL_, RV_DL_, and R_DL/NL_ accurately classified the control and COPD groups with AUCs greater than 0.93 and both sensitivity and specificity >88%. The mean AV/TV and PE_-910_ presented a moderate performance in differentiating the COPD from the control group with AUCs of 0.83 (sensitivity, 74.3%; specificity, 86.5%) and 0.91 (sensitivity, 75.7%; specificity, 88.9%), respectively. As the slice thickness increased to 5 mm, the AUC, sensitivity, and specificity of all image biomarkers decreased. The AUCs of the mean AV/TV, PE_-910_, and PE_-950_ decreased to 0.81 (sensitivity, 67.1%; specificity, 86.5%), 0.85 (sensitivity, 67.1%; specificity, 89.2%), and 0.88 (sensitivity, 72.9%; specificity, 89.2%), respectively. In contrast, the image biomarkers from the RAVHs maintained accurate classification. The AUCs of the RV_NL_, RV_DL_, and R_DL/NL_ from the 5-mm-thick images were 0.95 (sensitivity, 90.0%; specificity, 91.9%), 0.96 (sensitivity, 87.1%; specificity, 94.6%), and 0.97 (sensitivity, 87.1%; specificity, 97.3%), respectively.

**Table 2:** Diagnostic accuracy of image biomarkers of the mean AV/TV, PE_-910_, PE_-950_, RV_NL_, RV_DL_, and R_DL/NL_ from 2.5- and 5-mm-thick images.

Slice thickness	Image biomarker	AUC	Sensitivity (%)	Specificity (%)
2.5 mm	Mean AV/TV	0.83	74.3	86.5
	PE_-910_	0.91	75.7	88.9
	PE_-950_	0.94	88.6	96.3
	RV_NL_	0.98	94.3	97.3
	RV_DL_	0.99	90.0	100.0
	R_DL/NL_	0.99	92.9	97.3
5 mm	Mean AV/TV	0.81	67.1	86.5
	PE_-910_	0.85	67.1	89.2
	PE_-950_	0.88	72.9	89.2
	RV_NL_	0.95	90.0	91.9
	RV_DL_	0.96	87.1	94.6
	R_DL/NL_	0.97	87.1	97.3

## Discussion

Here, the utility of the TCM in calculating AV/TV from CT images and the application of AV/TV in COPD diagnosis were evaluated. The TCM can inherently nullify PVE resulting from insufficient spatial resolution of clinical CT images, thereby yielding an accurate AV/TV. Moreover, the RAVH calculated from AV/TV can describe the characteristics of the air content in lungs, which is useful for COPD diagnosis. Radiologists can detect areas with higher air content from the AV/TV maps and diagnose COPD using the RAVH.

Quantification analysis of CT images for COPD diagnosis and evaluation is based on the size of the low-attenuation area computed using a predefined threshold. However, the values and variations of the CT numbers of a tissue presented in CT images are dependent on the size of the tissue relative to that of the voxel owing to the averaging behavior of PVE. Consequently, the same tissue could present different CT numbers under different voxel sizes. The thresholds should be optimized for a particular image slice thickness to standardize the quantification results. However, the relationship between optimal thresholds and slice thickness remains unclear and difficult to determine. Moreover, the pixel size could influence the optimal thresholds. As noted in the phantom studies, the cavity volumes and foam masses can be overestimated by binary segmentation if the resolution of the CT images is insufficient to obtain a clear image of the air cavities and fine pores. By contrast, the TCM with the submaterial pairs identical to the phantoms was insensitive to slice thickness and could accurately calculate the cavity volumes and foam masses from the CT images, even with a 10-fold decrease in slice thickness. However, the foam phantom study indicated the numerical distribution of the CT numbers of the foams was influenced by the pore size relative to the voxel. The RV_98.75%_ from the RAVHs of the foams varied with the slice thickness when the pore structures can be captured by CT images. Otherwise, the foams in the CT images presented as uniform objects, and the RV_98.75%_ of the foams remained constant across different slice thicknesses.

Muscle and blood were used as the representative tissues and their CT numbers were applied for calculating AV/TV. The effect of the CT number of the representative tissue in calculating AV/TV was insignificant. It was mainly caused by the CT number of lungs usually being lower than −800 and closed to that of air (−1000). The CT number of air, which can be directly measured from CT images, thereby significantly affected AV/TV calculation. In addition, the tube voltage affects the measured linear attenuation coefficient of tissues, which is reflected in the subsequently calculated CT number. In the TCM, the imaging parameter of the tube voltage was implied in the measured CT numbers of the submaterials. Therefore, the AV/TV calculated using the TCM intrinsically prevented the effect of the tube voltage. However, the CT number measured using ROIs in the aorta or ventricle could be affected by the contrast medium, which could further bias the calculation of AV/TV with the air–blood pair. Therefore, the air–muscle pair was preferred for calculating the AV/TV using the TCM.

Although both the groups had similar mean AV/TV values, the RAVHs from both slice thicknesses presented significant differences between groups, particularly in the 81.25%–98.75% AV/TV bins. Compared to the RAVH of the control group, the RV in the 81.25%–91.25% AV/TV bins shifted to the RV in the 98.75% AV/TV bin of the RAVHs of the COPD group, indicating that the air content in lungs increased with the formation of small air spaces from several damaged alveoli. The RV_DL_ also clearly identified air trapping in patients with COPD. However, the difference between the mean AV/TVs values of the control and COPD groups was <5% on average, indicating similar mean CT numbers of the lungs in the control and COPD groups. This is mainly because of the compensation between the RVs in the 81.25%–91.25% AV/TV bins and the 98.75% AV/TV bin. As in the foam phantom study, the RAVHs and image biomarkers were affected by the slice thickness. In the 2.5-mm-thick images, the RV shift caused the RAVHs of the COPD group to differ from the normal distribution-resembling RAVHs of the control group. When the slice thickness increased to 5 mm, the spatial coverage of a voxel increased, resulting in the inclusion of the air-trapping area, collapse of alveoli and surrounding tissues in a single voxel, and an averaged CT number because of PVE; furthermore, the RV_DL_ in the RAVHs of the COPD group decreased. Consequently, the CT numbers of the low-attenuation areas increased because of averaging with the surrounding tissues. The RV_NL_ increased and the PE_-910_, PE_-950_, RV_DL_, and R_DL/NL_ decreased as the slice thickness increased. Moreover, the differences between the image biomarkers of the control and COPD groups decreased as the slice thickness increased owing to the averaging behavior of PVE. Nevertheless, the image biomarkers from the RAVHs remained distinct between the control and COPD groups. All image biomarkers except the mean AV/TV and PE_-910_ demonstrated high differentiability between the control and COPD groups. However, the AUCs decreased as the slice thickness increased because of the decrease in the difference between the image biomarkers of the control and COPD groups. Notably, PE_-950_ presented better diagnostic performance with higher AUCs, sensitivity, and specificity than did PE_-910_ across both slice thicknesses. However, a threshold higher than −950 is recommended to calculate the PE using 5-mm-thick images [15]. This inconsistency may stem from the fact that previous studies focused on quantification rather than diagnosis [9,10,13,14,19,23]. To obtain comparable PEs from different slice thicknesses, further studies are warranted to determine optimal thresholds. Compared with that with the PE, the calculation of AV/TV from CT images only required the mean CT numbers of the submaterials, which are insensitive to the slice thickness and can be directly measured. Furthermore, the diagnostic performance of the image biomarkers from the RAVHs was superior to that of the PEs, with the AUCs greater than 0.94 across both slice thicknesses. For better diagnosis of COPD, CT images thinner than 5 mm should be used.

The TCM has no hardware or imaging parameter requirements for calculating AV/TV from CT images. However, image artifacts could affect the measured CT numbers of lung tissues and bias the subsequently calculated AV/TV. Effective image artifact reduction methods should be applied before the calculation of AV/TV. In addition, statistical or iterative methods commonly used in the reconstruction of low-dose CT images for noise reduction could reduce the fluctuation of CT numbers and result in a highly concentrated RAVH from monotonous AV/TV values. The ability of the image biomarkers from such noise-reduced RAVHs to characterize the air content in lungs may decrease. The image biomarkers from the CT images with and without noise reduction techniques should be compared separately. Analogous to the PFT, the RAVH can characterize the air content in the patient’s lungs. The image biomarkers RV_NL_, RV_DL_, and R_DL/NL_ can help understand the RVs of normal and damaged lungs. In addition, a lung volume fraction can be simultaneously obtained from the calculation of the TCM. As in several recent studies, novel quantification methods or image biomarkers are proposed to estimate pulmonary function [29], classification of disease phenotype [30], and dynamic ventilation assessment [31] from CT images. In future studies, the RAVH will be applied to severity grading of COPD, assessing functional small airways diseases, and dynamic ventilation assessment. In addition, the lung volume fractions will be used to evaluate pulmonary fibrosis in interstitial pneumonia.

## Conclusion

In this study, a TCM was used to convert chest CT images to AV/TV maps, and image biomarkers calculated from the AV/TV maps were used to diagnose COPD. The TCM is unaffected by PVE, ensuring accuracy of the calculated AV/TV from the CT images with different voxel sizes. In addition, the TCM only requires a representative CT number of lung parenchyma for conversion. For COPD diagnosis, the image biomarkers of RAVH, RV_NL_, RV_DL_, and R_DL/NL_ calculated from the AV/TV maps present a high performance in differentiating patients with COPD from control participants, even on 5-mm-thick CT images. In conclusion, RAVH, RV_NL_, RV_DL_, and R_DL/NL_ calculated from chest CT images using the TCM are reliable image biomarkers for characterizing air content in lungs and diagnosing COPD.

## Supporting information

S1 Fig Appearance and dimensions of cavity phantoms with addressed septa widths and spacing. (TIF)

S2 Fig Binary segmentation results from Fig 3(A) using (a) the threshold of -950 and (b) the thresholds from Otsu’s method.
(TIF)

S3 Fig Bland–Altman plots of the differences between the mean AV/TV values calculated from (a) 2.5- and (b) 5-mm-thick images using the TCM with air–muscle and air–blood submaterial pairs. The mean difference (solid line) and the 95% limits of agreement (dashed line, ± 1.96 *σ*) are shown.
(TIF)

S4 Fig Boxplots of the image biomarkers of the (a) mean AV/TV, (b) PE_-910_, (c) PE_-950_, (d) RV_NL_, (e) RV_DL_, and (f) R_DL/NL_ of the control and COPD groups calculated from the 2.5- and 5-mm-thick CT images.
(TIF)

S1 Table Physical densities (*ρ*), electron densities (*N*_*g*_), and effective atomic numbers (*Z*_eff._) of the tissues commonly appearing in chest CT images, including lung parenchyma, aorta, blood, heart, and skeletal muscle. (DOCX)
