# Supplementary figures and images for: Calculating air volume fractions from computed tomography images for chronic obstructive pulmonary disease diagnosis
Source: PLoS One. 2020 Apr 16;15(4):e0231730. doi: 10.1371/journal.pone.0231730 (PMC7162278; doi:10.1371/journal.pone.0231730)

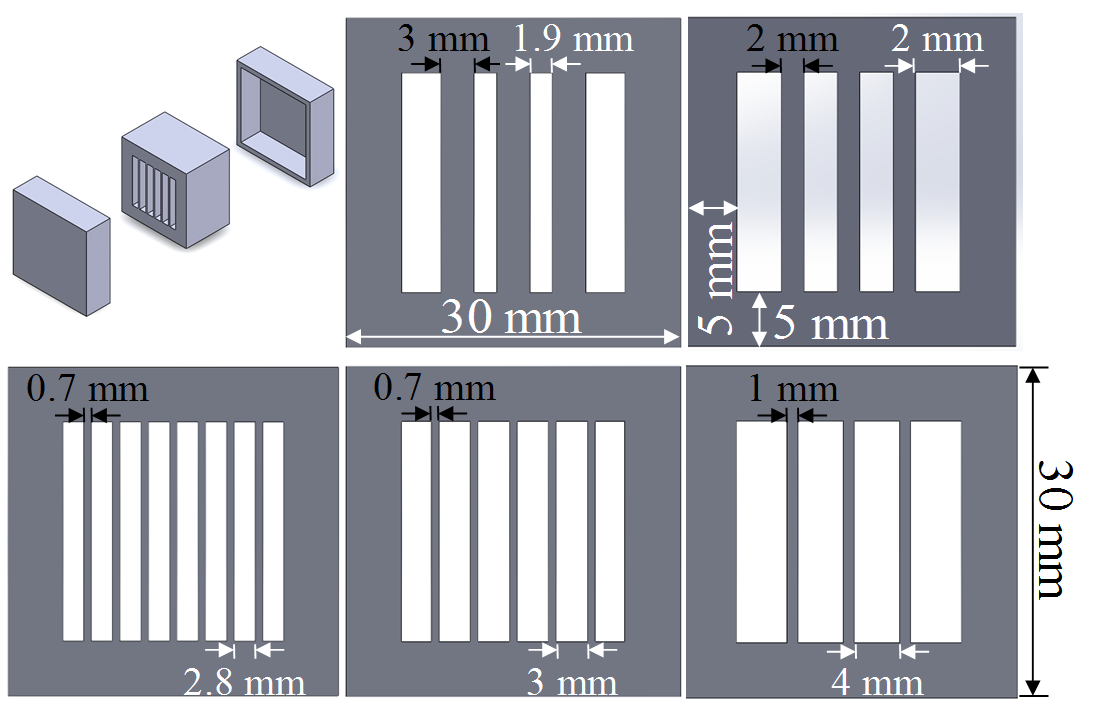

Supplement: S1 Fig — (TIF) [file pone.0231730.s001.tif]

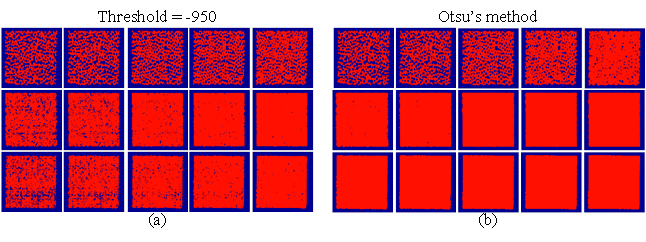

Supplement: S2 Fig — Binary segmentation results from Fig 3(A) using (a) the threshold of -950 and (b) the thresholds from Otsu’s method. (TIF) [file pone.0231730.s002.tif]

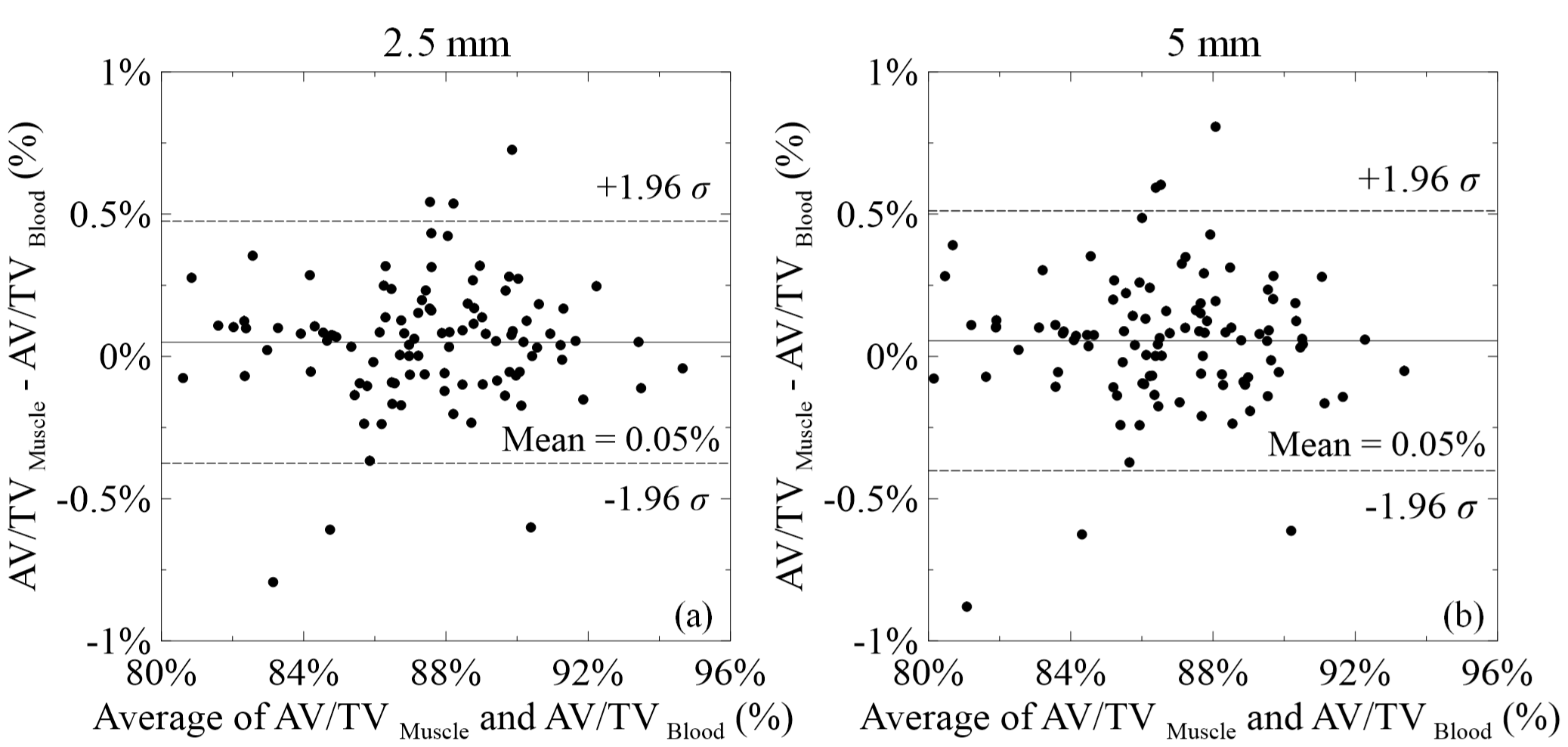

Supplement: S3 Fig — Bland–Altman plots of the differences between the mean AV/TV values calculated from (a) 2.5- and (b) 5-mm-thick images using the TCM with air–muscle and air–blood submaterial pairs. The mean difference (solid line) and the 95% limits of agreement (dashed line, ± 1.96 σ) are shown. (TIF) [file pone.0231730.s003.tif]

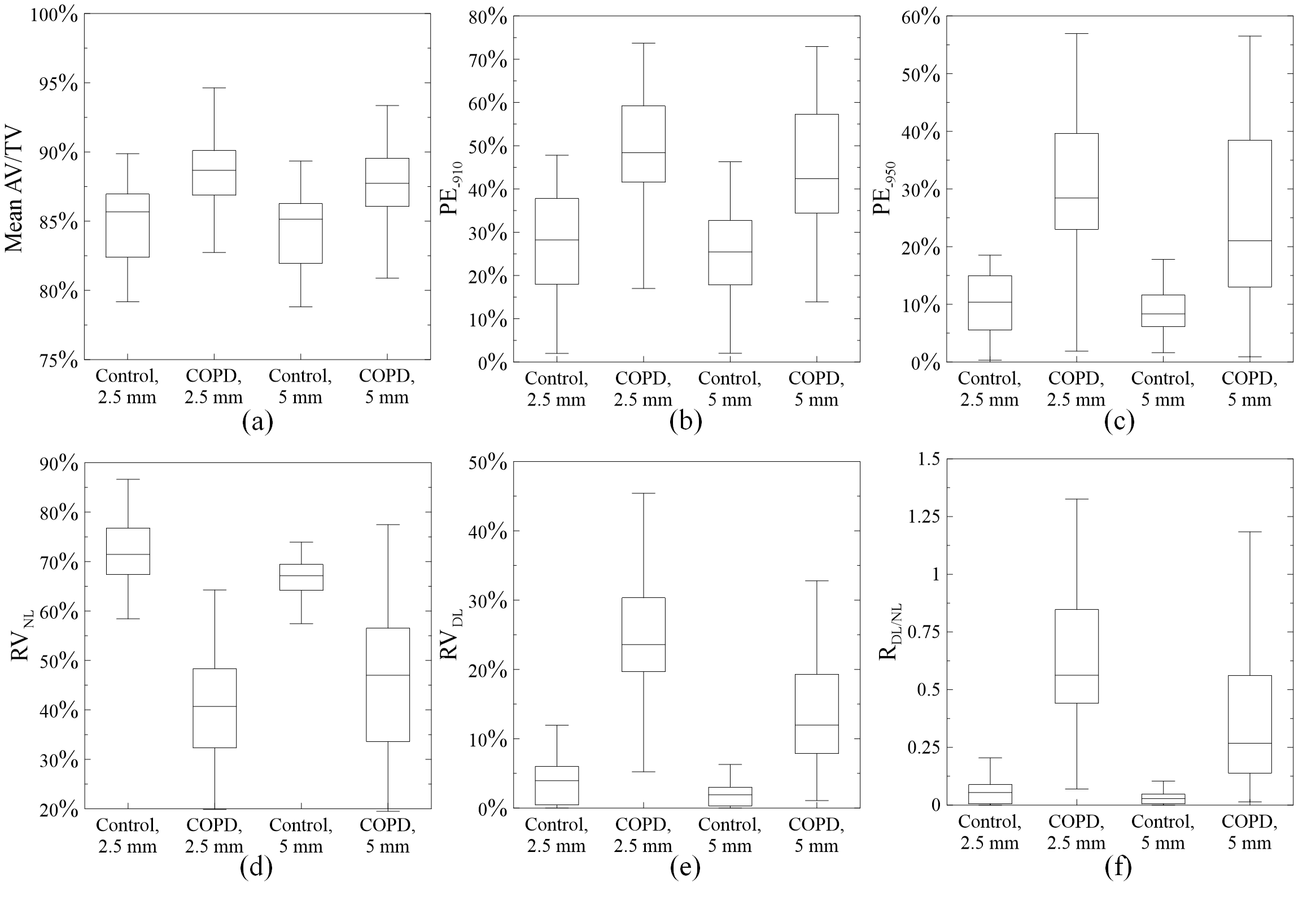

Supplement: S4 Fig — Boxplots of the image biomarkers of the (a) mean AV/TV, (b) PE-910, (c) PE-950, (d) RVNL, (e) RVDL, and (f) RDL/NL of the control and COPD groups calculated from the 2.5- and 5-mm-thick CT images. (TIF) [file pone.0231730.s004.tif]
