# Supplementary material for: Calculating air volume fractions from computed tomography images for chronic obstructive pulmonary disease diagnosis
Source: PLoS One. 2020 Apr 16;15(4):e0231730. doi: 10.1371/journal.pone.0231730 (PMC7162278; doi:10.1371/journal.pone.0231730)
Supplement: S1 Table — (DOCX) [file pone.0231730.s005.docx]

S1 Table. Physical densities (*ρ*), electron densities (*N_g_*), and effective atomic numbers (*Z*_eff._) of the tissues commonly appearing in chest CT images, including lung parenchyma, aorta, blood, heart, and skeletal muscle.

| Tissue | *ρ* (g cm^-3^) | *N_g_* (*e* cm^-3^) | *Z*_eff._ |
| --- | --- | --- | --- |
| Lung parenchyma | 1.05 | 3.31×10^23^ | 7.40 |
| Aorta | 1.05 | 3.30×10^23^ | 7.43 |
| Blood, whole | 1.06 | 3.31×10^23^ | 7.44 |
| Heart, 1 | 1.05 | 3.31×10^23^ | 7.27 |
| Heart, 2 | 1.05 | 3.32×10^23^ | 7.32 |
| Heart, 3 | 1.05 | 3.32×10^23^ | 7.38 |
| Heart, blood filled | 1.06 | 3.31×10^23^ | 7.42 |
| Muscle, skeletal, 1 | 1.05 | 3.31×10^23^ | 7.31 |
| Muscle, skeletal, 2 | 1.05 | 3.31×10^23^ | 7.35 |
| Muscle, skeletal, 3 | 1.05 | 3.31×10^23^ |  |
